# Impact of hospital complaint handling on promoting high-quality development of hospitals via an emotional language analysis model: a case study of a tertiary hospital service center in Quanzhou city, Fujian province

**DOI:** 10.3389/frhs.2025.1610004

**Published:** 2025-08-26

**Authors:** Caijiao Zheng, Yi Zhang, Xiaolong Lian, Jinxiu Ke, Hongxia Chen, Yiwen Chen

**Affiliations:** ^1^ Department of Outpatient, Quanzhou First Hospital, Quanzhou, Fujian, China; ^2^ Office of the President, Quanzhou First Hospital, Quanzhou, Fujian, China; ^3^ Department of Medical Affairs, Quanzhou First Hospital, Quanzhou, Fujian, China

**Keywords:** patient complaint analysis, hospital service quality, data cleaning, DISC behavioral language model, KANN-DBSCAN clustering model

## Abstract

**Background:**

In the healthcare service industry, patient complaints serve not only as a critical metric for assessing hospital service quality but also as a fundamental driver of high-quality hospital development. Through a systematic analysis of patients' perceptions, opinions, and emotional responses to hospital management within the complaint-handling process.

**Methods:**

Therefore, this paper aims to develop a hospital complaint-handling analysis model to enhance public satisfaction with greater precision. First, complaint data from hospitals spanning January to December 2022–2024 was preprocessed using data cleaning, mechanical compression, word segmentation, and stop-word filtering techniques. Second, the DISC behavioral language model was employed to analyze key indicators, including hospital compensation frequency, total compensation amounts, patient appeal rates, complainants' satisfaction with the resolution process, and their overall satisfaction with complaint outcomes. Finally, a sentiment analysis model and an improved KANN-DBSCAN clustering model were applied to complaint data to precisely identify sentiment-related keywords and assess the intensity of negative emotions, providing hospitals with targeted improvement recommendations.

**Results:**

This study applied the DISC behavioral model to medical complaints. DISC-based text analysis enabled tailored responses. Among 334 intervention and 341 control cases, satisfaction 93.39%, was higher in the intervention group 83.24%, indicating improved complaint resolution through behavior-informed communication strategies.

**Conclusions:**

By analyzing patients' psychological needs and expectations, this study aims to minimize financial compensation and reduce patient appeals while enhancing overall complaint resolution satisfaction, which provides medical institutions with a more comprehensive, effective, and personalized complaint-handling strategy while simultaneously improving patients' healthcare experiences.

## 1 Introduction

In the healthcare service industry, patient complaints serve as a critical indicator of hospital service quality and a fundamental driver of high-quality hospital health care and service deliver (1–3). With advancements in medical technology and increased public awareness of health, expectations of hospitals have shifted from solely focusing on medical expertise to emphasizing service experience, doctor-patient communication, and treatment efficiency (4). Complaint data reflect patients' genuine concerns and highlight weaknesses in hospital management (5–7). If effectively analyzed and addressed, this information can help optimize medical procedures, enhance service quality, and improve patient satisfaction, ultimately contributing to hospital health care and service deliver.

In today's healthcare environment, hospitals are witnessing a continuous rise in complaints, which are becoming increasingly diverse in origin, complex in nature, and extensive in impact. Patient complaints not only create distress for patients and their families but also impose financial and reputational risks on healthcare institutions (8). To safeguard the interests of both patients and healthcare providers, fostering a harmonious doctor-patient relationship is imperative (9). Complaint resolution has become a crucial challenge for medical institutions, yet traditional approaches, which primarily rely on manual review and experiential judgment, are often inefficient and fail to systematically capture the emotional tendencies and core concerns embedded in complaints. Moreover, patient emotions and expectations are often conveyed in free-text complaints, making it difficult for conventional quantitative analysis to fully grasp their deeper meanings (10). Consequently, applying advanced information technologies to systematically analyze large-scale patient complaint data, precisely identifying emotional tendencies and extracting key concerns, has become an important research direction for improving hospital service quality (11).

In China, tertiary hospitals play a pivotal role in providing comprehensive healthcare services and are subject to stringent quality assurance mechanisms (12). These mechanisms typically encompass multiple dimensions, including patient safety, service efficiency, and patient-centered care. Patient complaints are regarded as critical indicators for evaluating hospital service quality, as they often reflect gaps in communication, care processes, or service delivery. Standard quality assurance frameworks employ key performance indicators (KPIs), such as complaint resolution timeliness, compensation rates, patient satisfaction scores, and recurrence of complaints, to monitor and improve service standards (13). By systematically analyzing patient complaints within these frameworks, hospitals can identify systemic deficiencies, implement targeted interventions, and foster continuous improvement in service quality and patient trust.

**Figure 1:**
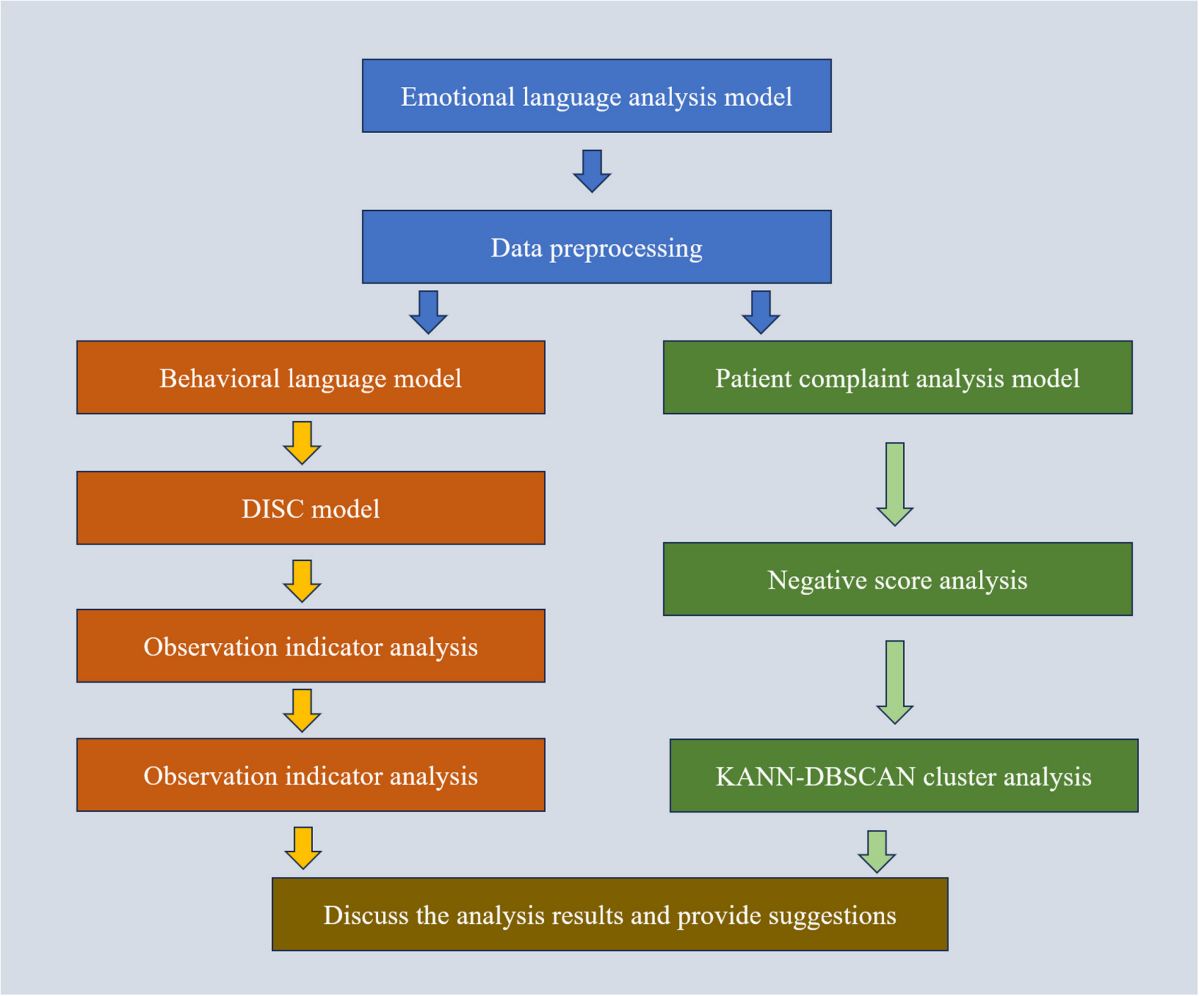
Proposed model flowchart. The preprocessed data is analyzed through Behavioral Language Model, DISC Model, and Observation Indicator Analysis Patient complaint analysis model, Negative score analysis and KANN-DBSCAN cluster analysis Analyze the results to obtain the final analysis results and provide suggestions together.

The DISC (Dominance, Inducement, Submission, and Compliance) behavioral language model offers a theoretical framework for targeted intervention in complaint resolution by considering complainants' personality traits and emotional responses (14). Integrating this theoretical model into hospital complaint-handling processes can help address the emotional needs of both patients and healthcare providers, facilitating equitable, rational, and legally compliant dispute resolution. In the era of rapid technological advancement, hospitals must innovate with their complaint-handling mechanisms, adapting complaint management strategies to align with evolving healthcare expectations (15). Through systematic complaint analysis, hospitals can gain valuable insights into public opinion trends related to their services, allowing them to enhance service quality and bolster their competitive position (Figure 1).

Effective management of patient complaints requires both personalized communication strategies and accurate identification of factors leading to dissatisfaction. The DISC behavioral language model enables tailoring responses based on patients' communication styles, aiming to rebuild trust and enhance satisfaction. However, understanding the root causes of dissatisfaction remains challenging due to the unstructured nature of complaint data. To address this, we combined DBSCAN with KANN-DBSCAN, an improved density-based clustering algorithm with enhanced similarity measurement, to better categorize complaints and reveal underlying issues. This study aimed to evaluate whether integrating the DISC model with advanced clustering methods could improve complaint resolution, reduce patient conflict, and provide actionable insights into service quality improvement. Therefore, in this paper, taking the complaint data obtained from a tertiary hospital in Quanzhou, Fujian as an example, thereby identifying deficiencies across multiple service dimensions. By leveraging this model, hospital administrators can accurately capture patients' core concerns, refine service workflows, and improve doctor-patient relationships, ultimately facilitating a transition toward more efficient, patient-centered, and intelligent hospital operations.

## 2 Materials and methods

### 2.1 Data

The data for this study were collected from a tertiary hospital service center in Quanzhou, Fujian Province, encompassing 1,278 hospital complaints from January 2022 to December 2024. This study was conducted at a tertiary teaching hospital in Quanzhou, Fujian Province, China. The hospital was selected as the study setting because it serves as one of the largest comprehensive healthcare centers in the region, with a high annual patient volume and a well-established quality assurance system. It also operates an advanced complaint management platform that systematically records patient feedback, making it particularly suitable for analyzing complaint patterns and evaluating communication models. Furthermore, the authors are employed at this hospital, ensuring direct access to the complaint management system and guaranteeing the validity and completeness of the data collected. The dataset includes both internal and external hospital complaints. Internal complaints primarily originated from in-person visits and telephone complaints received by the hospital service center, amounting to 675 cases. External complaints were grievances forwarded by higher administrative authorities and the 12,320 hotlines, comprising 603 cases, and randomly select 1,500 follow-up data from 2023–2024, respectively. To uphold complainants' privacy, all personally identifiable information was meticulously handled to safeguard their rights.

Preliminary data analysis identified missing values in certain attributes and encoding errors in attribute names. To mitigate potential impacts on subsequent analyses, initial data pre-processing was conducted. Given the low proportion of missing complaint content, the affected records were excluded. The raw dataset exhibited inconsistencies in time-related data; therefore, erroneous date values were replaced with “—” (15–17).

The dataset was divided into two groups: complaints recorded from January to December 2022–2023 formed the control group (*n* = 334), whereas those from January to December 2024 constituted the intervention group (*n* = 341). The control group included 112males and 222 females, aged 18–74 years (mean age: 39.88 ± 2.57years). The intervention group consisted of 138 males and 203 females, aged 17–81 years (mean age: 40.21 ± 2.45 years). Statistical comparisons of baseline characteristics between the two groups revealed no significant differences (*P* > 0.05), confirming their comparability. Informed consent was obtained from all participating patients and their families. Ethical approval for this study was granted by the hospital's institutional review board.

### 2.2 Group allocation and data selection

#### 2.2.1 Assessment of randomization

Baseline Balance Assessment. Key baseline variables, such as complaint type and complaint date, were compared between groups to evaluate the balance and verify the success of randomization.

Documentation of the Randomization Process. The randomization procedure was rigorously recorded, including details of the random allocation method (e.g., computer-generated random numbers, block randomization), to ensure that the assignment process was both unpredictable and free from investigator bias.

#### 2.2.2 Data selection criteria

To ensure the validity and reliability of the study findings, the following data selection criteria were applied:

**Inclusion Criteria.** Timeframe: complaints filed between January 2022 and December 2024; Complaint type: only complaints related to healthcare service quality were included; Data completeness: only records with complete information, including satisfaction ratings, were retained.

**Exclusion Criteria.** Records lacking essential data (e.g., missing complaint date or satisfaction score) were excluded from the analysis.

**Sample Size Estimation.** A power analysis was conducted based on the expected effect size to ensure that both the control and intervention groups had adequate sample sizes to detect statistically significant differences.

**Temporal Matching.** To minimize potential bias due to timing, the distribution of complaint dates was aligned between the two groups to ensure comparability.

### 2.3 Behavioral language model

The control group followed the standard complaint handling process: receiving complaints → registering issues → addressing concerns → providing feedback (18–21). The intervention group utilized the DISC behavioral language model to process complaints, building upon the standard procedure. The approach involved raising awareness, shifting service perspectives, and responding promptly to complaints. At the initial stage, a proactive and positive approach was adopted, with courteous greetings such as “Hello! How can I assist you?” to help ease patients' emotions and establish effective communication (22–25).

During the complaint process, patients were often highly emotional, requiring an outlet to vent their dissatisfaction. Staff actively listened and analyzed the complainant's language, tone, and body language using the DISC behavioral language model to assess their personality traits and respond accordingly. The complainant's psychological state, including the need to vent, desire for respect, and expectations for compensation, was considered to allow appropriate expression of dissatisfaction while consistently demonstrating respect (26). Based on the complainant's traits, a tailored complaint-handling approach and solutions were developed to ensure a refined management process, as shown in Table 1.

**Table 1:** Specific methods for handling patient complaints using the DISC behavioral language model theory. By analyzing the emotional type of the patient, analyzing their text and potential text, clarifying their purpose, and proposing corresponding solutions.

Type	Text	Subtext	Purpose	Correct handling
Patients with D-type trait (dominant type)	Merciless words	I only talk to the highest responsible person	Release emotions and solve problems	Responses such as “Thank you for your suggestion,” “Your feedback is highly valuable,” and “We will implement immediate improvements” can be effective.
I-type Personality (Influential Type) Patients	Tend to attract the attention of those around them with a loud voice.	“The issue I am reporting is very serious and must receive high attention.”	Express emotions to gain attention.	Show willingness to listen, sincerely consider the patient's opinions, and respond actively.
S-type Personality (Steady Type) Patients	Their communication style resembles a request for help, such as: “Could you please come and take a look?”	"I am experiencing difficulties and challenges here."	Express concern and seek assistance.	Acknowledge mistakes, offer an apology, and reassure the patient that their issue will be taken seriously.
C-type Personality (Compliant Type) Patients	Speak in a relatively reserved manner, saying things like: “Please provide me with a reasonable explanation.”	"I would like to understand why this issue has occurred.”	Seek clarification and offer suggestions.	Their inquiries are based on a desire to provide constructive feedback. They seek “facts” and expect a well-structured explanation.

Following the resolution of complaints, satisfaction surveys were administered on-site or through follow-up interviews via telephone, WeChat, or other methods. These surveys collected feedback regarding the handling of the issue, timeliness of resolution, and overall experience. Feedback was also used to monitor service quality, measure customer satisfaction, and implement necessary improvements in future service delivery (27–29).

Data on hospital compensation rate, compensation amount, and patient petition rate were compared between the two groups. An internally designed questionnaire, validated by experts, was used for evaluation. The standardized Cronbach's *α* coefficient of the questionnaire was 0.897, indicating good reliability. The survey questions included: “Are you satisfied with the reception by staff?”; “Are you satisfied with the complaint handling process?”; “Are you satisfied with the staff's attitude during the complaint process?”; “Was your complaint addressed and responded to in a timely manner?”; and “Are you satisfied with the hospital's resolution of your complaint?”. Each aspect included three options: “satisfied,” “generally satisfied,” and “dissatisfied.” Overall satisfaction = (number of satisfied + generally satisfied responses)/total number of responses × 100%.

Data analysis was performed using SPSS 22.0 statistical software. Continuous data are presented as mean ± standard deviation (x ± s), and group comparisons were performed using the independent two-sample *t*-test. Categorical data are presented as *n* (%), and comparisons were made using the chi-square (X²) test. A *p*-value of <0.05 was considered statistically significant (30, 31).

### 2.4 Patients' complaint analysis model

We selected the publicly available sentiment lexicon provided by Boson. Each word in the tokenized patients' comments was compared with the public sentiment lexicon. If the word exists in the lexicon, it is assigned the corresponding score from the lexicon (32). If the word does not have a corresponding entry in the lexicon, its score is set to 0, as shown in the following formula:

$$score_{n}=\{\begin{array}{c} z_{n},x_{n}=z_{n}\\ 0,x_{n}\neq z_{n} \end{array}$$

After comparing each token in the sentence or paragraph with the entries in the public sentiment lexicon, the cumulative score is considered the final sentiment score of the comment. If the resulting score is greater than 0, the comment is classified as having a positive sentiment. If the score is negative, it is classified as having a negative sentiment. If the score is 0, the sentiment is considered neutral. This score is used to assess the intensity of the patient's negative sentiment.

$$score=score_{1}+score_{2}+\ldots +score_{n}$$

**Figure 2:**
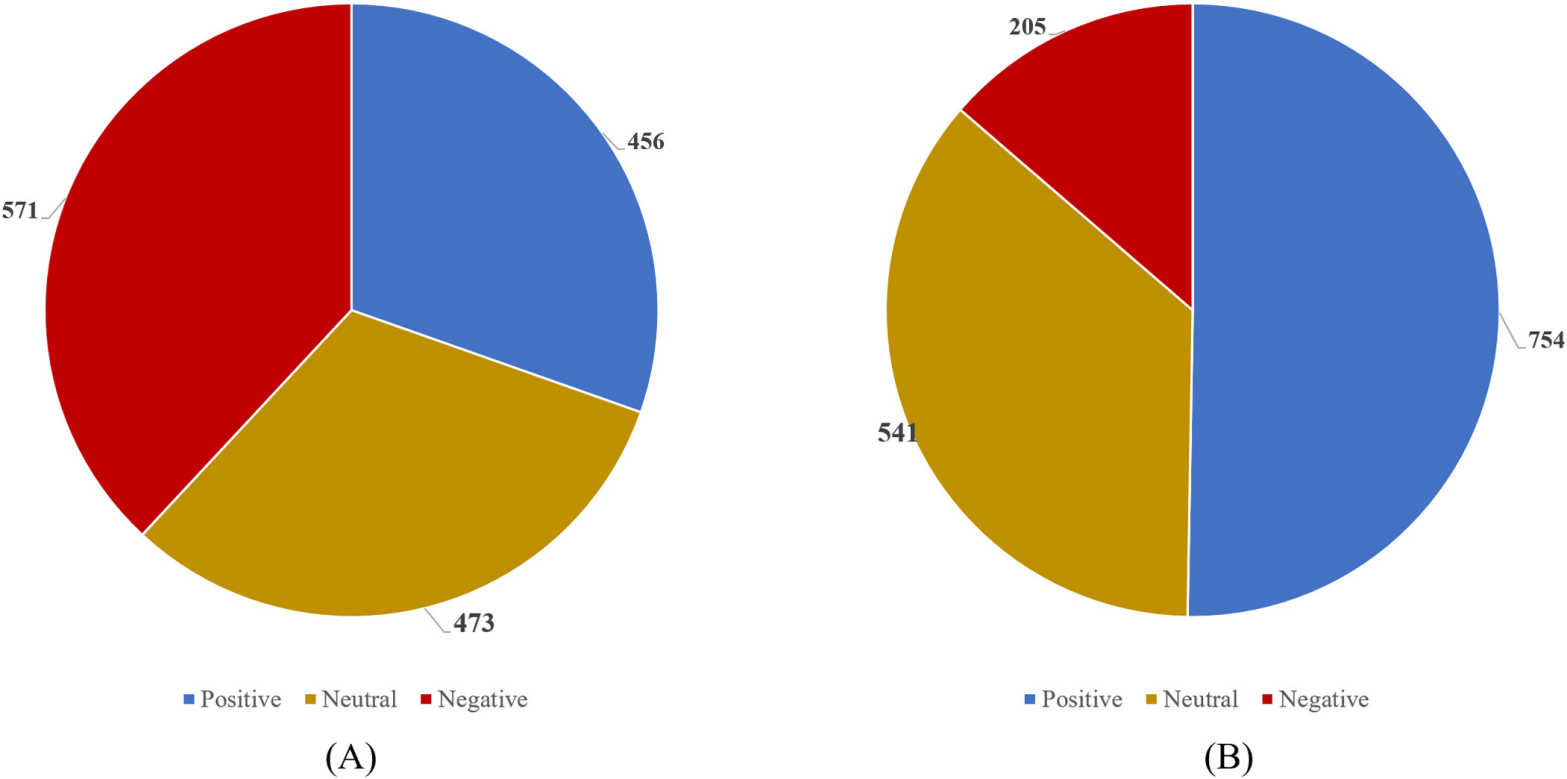
Distribution chart of positive (>0), negative (<0), and neutral emotions (=0) in 2023 and 2024 follow-up data. **(A)** 2023 Follow up Data. **(B)** 2024 Follow up Data.

We then score the follow-up data of users in 2023 and 2024 to obtain their attitude distribution, as shown in Figure 2. The DBSCAN clustering method was employed to analyze patients' complaints and identify specific categories contributing to lower patients' satisfaction. DBSCAN is a density-based clustering algorithm that performs well on low-dimensional data (24). However, the algorithm is highly sensitive to the selection of the *ε* and *P_min_* parameters. Inappropriate parameter selection may lead to suboptimal clustering performance or even incorrect results. As an improvement over DBSCAN, KANN-DBSCAN introduces a novel distance measurement method, which enables a more precise calculation of the similarity between data points. The computed similarity scores are then incorporated into the DBSCAN clustering process. Additionally, it mitigates errors caused by insufficient sample data. The steps of the KANN-DBSCAN algorithm are as follows:

$$D_{i,j}=\{Dist(i,j)\}$$

*D_n_* _×_ _n_ denotes an *n* *×* *n* times real symmetric matrix, where *n* denotes the number of objects contained in the dataset *D*. *Dist*(*i,j*) represents the distance between the *i* th and *j* th objects in dataset *D*. Next, each row of the distance matrix *D_n_* *_×_* *_n_* is sorted in ascending order. The first column of the sorted matrix forms the distance vector *D*_0_, which represents the distance of each object to itself and is uniformly zero. The *K* th column consists of the *K*-nearest neighbor distance vector *D_K_* for all data points. An *ε* parameter list is generated by computing the average of the elements in vector *D*_K_, yielding the K-mean nearest neighbor distance vector *D*_K_, which serves as a candidate for the *ε* parameter. By computing for all values of *K*, the parameter list *D_ε_* is obtained:

$$D_{\varepsilon }=\{\bar{D_{k}}\;\;\;|1\leq k\leq n|\}$$

*D_ε_* denotes the parameter list, and *D*_K_ denotes the nearest neighbor distance vector. The *P*_min_ list is generated using the mathematical expectation method. For a given *ε* parameter list, the number of objects in the *ε*-neighborhood is sequentially determined for each *ε* parameter. The mathematical expectation of the *ε*-neighborhood object count for all objects is then computed and used as the neighborhood density threshold parameter *P*_min_ for dataset *D*:

$$P_{\min}=\frac{1}{n}\overset{n}{\underset{i=1}{\sum }}\;P_{i}$$

*P*_min_ denotes the neighborhood density threshold, and *P*_i_ denotes the value of each threshold. The *ε* and *P*_min_ parameters are input into the DBSCAN algorithm for clustering analysis, generating cluster numbers corresponding to different *K* values. When the number of generated clusters remains consistent for three consecutive iterations, the clustering results are considered stable, and the corresponding *ε* and *P*_min_ values are regarded as the optimal clustering parameters. Through iterative computations, the process continues until the number of generated clusters deviates from *N*, at which point the maximum *K* value corresponding to *N* clusters is selected as the optimal *K* value. The optimal *K* value corresponds to the K-mean nearest neighbor distance *D*_K_, which is identified as the optimal *ε* parameter. Similarly, the *P*_min_ parameter corresponding to the optimal *K* value is determined as the optimal *P*_min_ parameter.

### 2.5 Implement

The KANN-DBSCAN model is implemented in Python 3.10, utilizing scikit-learn, numpy, pandas and matplotlib libraries. Prior to clustering, raw input data were standardized to zero mean and unit variance using the StandardScaler utility from scikit-learn. For high-dimensional datasets, optional dimensionality reduction was applied via Principal Component Analysis (PCA) to retain local structure while mitigating noise. For the k-approximate nearest neighbor step, we fix k = 20 as a balance between local density sensitivity and global structure preservation. And the *ε* value was determined via k-distance graph analysis, *ε* = 0.5 yielded stable results. All experiments were conducted on a machine running Windows 10 with an Intel i7 CPU and 8 GB RAM.

## 3 Results

### 3.1 Results of behavioral language model

**Analysis results of the hospital's compensation rate, compensation amount, and patient petition rate for complaint incidents.** A comparison of the compensation rates and petition rates between the two groups revealed no statistically significant differences (*P* > 0.05). However, the compensation amount in the experimental group was significantly lower than that in the control group, with a statistically significant difference (*P* < 0.05), as shown in Table 2.

**Table 2:** Comparison of hospital compensation rate, compensation amount, and patient petition rate between the application group and the control group for complaint events.

Group	Compensation rate [Example (%)]	Petition rate [Example (%)]
Control group (*n* = 161)	27 (8.15)	51 (15.32)
Application group (*n* = 198)	12 (3.56)	9 (2.49)
X^2^/t value	6.324	30.561
*P* value	0.012	<0.001

**Comparison of Complainants' Satisfaction with the Handling Results of Complaint Incidents.** The satisfaction levels of complainants in the experimental group regarding hospital complaint reception, complaint handling procedures, staff service attitudes, response timeliness, and final outcomes were significantly higher than those in the control group, with a statistically significant difference (*P* < 0.05), as shown in Table 3.

**Table 3:** Comparison of satisfaction with complaint handling results between application group and control group complainants [example (%)].

Group	Upon receiving your complaint, were you satisfied with the staff's reception?	Were you satisfied with the complaint-handling process conducted by the staff?	Were you satisfied with the complaint-handling process conducted by the staff?	Did the hospital respond to your complaint and suggestions in a timely manner?	Were you satisfied with the hospital's resolution of your complaint?
Control group (*n* = 161)	307 (77.92)	262 (79.01)	291 (78.73)	324 (93.16)	260 (78.81)
Application group (*n* = 198)	328 (90.38)	339 (92.71)	329 (92.06)	340 (99.13)	316 (93.83)
X^2^ value	22.41	28.67	26.83	15.32	33.15
*P* value	<0.001	<0.001	<0.001	<0.001	<0.001

**Analysis of Complainants' Overall Satisfaction with the Handling Outcome.** The overall satisfaction of complainants in the experimental group was significantly higher than that in the control group, with a statistically significant difference (*P* < 0.05), as shown in Table 4.

**Table 4:** Comparison of overall satisfaction of complainants with the handling results between the application group and the control group [example (%)].

Group	Satisfied	Generally satisfied	Dissatisfied	Total satisfaction
Control group (*n* = 161)	85 (25.43)	193 (57.82)	56 (16.87)	278 (83.24)
Application group (*n* = 198)	143 (41.97)	23 (51.34)	23 (6.75)	318 (93.39)
X^2^ value	-	-	-	16.45
*P* value	-	-	-	<0.001

### 3.2 Results of patients' complaint analysis model

The data results obtained from the above analysis are shown in Table 5. This paper conducts clustering for the two control groups and analyzes their key terms, offering recommendations for improving the hospital's public service quality.

**Table 5:** Analysis results of patient complaint analysis model. The higher the negative score, the deeper the patient's negative emotions.

Group	Negative score	Keywords obtained from clustering
2023	−17	“Poor attitude”, “poor service”, and “poor environment”
2024	−8	“Negligence, lack of timeliness”, “tardiness”, and “failure to explain clearly”

### 3.3 Analysis of model results

#### 3.3.1 Analysis of behavioral language model results

The study found that there was no statistically significant difference in the compensation rate and petition rate between the two groups (*P* > 0.05). However, the compensation amount in the experimental group was significantly lower than in the control group, with a statistically significant difference (*P* < 0.05). The overall satisfaction of complainants in the experimental group was significantly higher than that in the control group, with a statistically significant difference (*P* < 0.05).

Univariate analysis revealed that the satisfaction rate was significantly higher in the intervention group (93.38%) compared to the control group (83.24%). A Shapiro–Wilk test confirmed that satisfaction scores in both groups followed a normal distribution (intervention group: W = 0.978, *p* = 0.102; control group: W = 0.981, *p* = 0.134), justifying the use of parametric tests.

Furthermore, after adjusting for potential confounding variables using multivariable logistic regression, the intervention group remained significantly associated with higher satisfaction, as shown in Table 6. The adjusted odds ratio (aOR) was 3.06 (95% CI: 1.66–5.64), indicating an independent effect of the DISC model on improving satisfaction. Sensitivity analyses further confirmed the robustness of this association.

**Table 6:** Multivariable logistic regression analysis results (*n* = 1,278). The multivariable logistic regression model examined the association between the intervention and satisfaction, adjusting for age, complaint source, and complaint type.

Variable	*β* (SE)	Adjusted OR (95% CI)	*P*-value
Intervention	1.12 (0.31)	3.06 (1.66–5.64)	<0.001
Age (per year)	–0.02 (0.01)	0.98 (0.96–1.00)	0.073
External Complaint	–0.45 (0.28)	0.64 (0.37–1.10)	0.108
Technical Complaint	–0.67 (0.32)	0.51 (0.27–0.96)	0.037

By understanding their behavioral patterns, healthcare providers can better comprehend their needs and expectations, leading to more effective problem resolution. Furthermore, healthcare institutions can tailor complaint handling processes based on the individual characteristics of the complainants. By understanding their behavioral patterns, healthcare providers can better comprehend their needs and expectations, leading to more effective problem resolution. Furthermore, healthcare institutions can tailor complaint handling processes based on the individual characteristics of the complainants This demonstrates that applying the DISC behavioral language model theory to medical complaints allows for a deeper analysis of complainants' behavior and emotions. By understanding their behavioral patterns, healthcare providers can better comprehend their needs and expectations, leading to more effective problem resolution. Furthermore, healthcare institutions can tailor complaint handling processes based on the individual characteristics of the complainants (32–34).

For instance, complainants with a dominant (D-type) personality tend to seek control and authority. These individuals require healthcare providers to demonstrate professional knowledge and problem-solving skills, while also showing deep concern for the patient.

On the other hand, complainants with an influential (I-type) personality value social interaction and emotional exchange. To communicate effectively with these individuals, it is necessary to establish a friendly and open environment, listen to their concerns, and provide timely feedback (35).

For steady (S-type) complainants, who tend to be introverted and avoid conflict, it is important to show patience, understand their feelings, and create a safe communication space.

Lastly, compliant (C-type) complainants require professionalism, strong responsibility, and adherence to rules and procedures. Providing efficient and precise solutions while ensuring everything is conducted according to established protocols is essential to meeting their expectations and earning their trust (36).

In addition to analyzing behavior and emotions, the DISC model can be applied to improve healthcare institutions can create a warm and comfortable environment, deliver meticulous services, and provide refined medical techniques, offering a unique healthcare experience. By understanding the needs of patients with different behavioral types, healthcare institutions can create a warm and comfortable environment, deliver meticulous services, and provide refined medical techniques, offering a unique healthcare experience.

#### 3.3.2 Analysis of patients' complaint analysis model results

Compared to 2023, the key terms in patients' complaints for 2024 have notably shifted from “poor attitude, poor service, and poor environment” to “negligence, untimeliness, lateness, and lack of clear explanation.” Meanwhile, the negative emotion score decreased from −17 to −8, indicating improvements in service attitude and environment, though issues related to medical efficiency and communication persist. In 2023, complaints primarily focused on healthcare staff's service attitude, overall service quality, and the hospital environment. These issues may have been caused by poor communication between medical staff and patients, inadequate staff training, or environmental factors that affected patient experience (37).

By 2024, the focus of complaints shifted towards issues in the medical process, such as delayed responses to patient needs, long waiting times, and unclear communication. This suggests that patient satisfaction with basic services has improved, but there are now higher expectations regarding medical efficiency and communication quality.

To address these changes, the hospital can implement the following measures to further enhance service quality. First, optimize personnel scheduling to ensure reasonable shifts for medical staff, reducing issues of tardiness and slow response times. Additionally, an appointment reminder system can be introduced to minimize delays caused by negligence or inefficient processes. Second, strengthen communication training for healthcare staff, encouraging proactive engagement with patients to ensure that medical processes and treatment plans are clearly explained, thereby preventing misunderstandings caused by information asymmetry. Furthermore, the hospital could implement electronic medical records and intelligent consultation systems to improve information transmission efficiency, allowing patients to more easily access medical information and appointment scheduling. Finally, although environmental issues were not prominent in 2024 complaints, it is still important to maintain cleanliness and optimize the waiting experience to consolidate the improvements.

Overall, while the hospital's services have improved, continuous efforts are needed to enhance medical efficiency, optimize communication methods, and refine service processes to further reduce negative emotions and improve patient experience and satisfaction.

## 4 Limitations

While the KANN-DBSCAN model demonstrates promising performance in clustering high-dimensional data with improved computational efficiency, several limitations should be acknowledged to contextualize the findings of this study.

### 4.1 Limitations of the clustering method

Although DBSCAN is effective for detecting clusters of arbitrary shape and handling noise, it has several known limitations:

**Sensitivity to Parameters**: The performance of DBSCAN is highly sensitive to the selection of *ε* and *P*_min_. While the k-distance graph method was used to guide parameter selection, it remains heuristic in nature, and may yield suboptimal clustering in datasets with varying density.

**Scalability to Very Large Datasets**: While FAISS enhances the speed of neighbor search, the density-based clustering stage itself (DBSCAN) does not scale linearly and may become computationally intensive for very large datasets (e.g., >10^6^ points).

**Inability to Handle Overlapping Clusters**: DBSCAN may struggle when clusters overlap significantly or when there is insufficient contrast between high- and low-density regions.

To address these issues, future research could explore adaptive density clustering algorithms or integrate hybrid approaches such as HDBSCAN, OPTICS, or neural density estimators that account for varying cluster densities more robustly.

### 4.2 Potential bias in human-centric or clinical applications

In applications involving patient data or subjective input (e.g., symptoms, survey responses), there exists a risk of **perception bias** or **self-reporting inconsistencies** that can affect the input features used for clustering. The current study does not incorporate measures to mitigate such bias (e.g., weighting features, incorporating uncertainty estimates), which may impact the interpretability or clinical relevance of the discovered clusters.

Further research should investigate how to incorporate data provenance, uncertainty modeling, or longitudinal feedback into the clustering pipeline to improve reliability in decision-critical environments.

### 4.3 Lack of interpretability in cluster structure

Another important consideration is the interpretability of the resulting clusters, especially when applied to domains requiring transparency (e.g., healthcare). While the KANN-DBSCAN model efficiently groups data based on density, it does not provide intrinsic explanations for cluster membership or feature importance.

Future work could integrate explainable AI (XAI) methods or *post-hoc* interpretability techniques (e.g., SHAP, LIME) to better understand the defining characteristics of each cluster and support stakeholder trust in the model outputs.

## 5 Discussion

This study demonstrates that integrating the DISC behavioral language model with advanced clustering methods (DBSCAN enhanced via KANN-DBSCAN) significantly improves patient complaint handling and satisfaction outcomes. Our results corroborate prior findings that behavioral communication frameworks can enhance healthcare interactions by enabling staff to tailor responses to patients' communication styles (38, 39). However, unlike earlier works that examined communication or complaint analytics in isolation (40), our approach uniquely combines DISC-guided personalization with data-driven clustering, allowing both the identification of latent complaint patterns and real-time adjustment of communication strategies. Notably, we discovered previously unrecognized complaint categories closely associated with specific DISC profiles, offering actionable insights for refining service workflows and reducing conflict escalation.

The observed improvement in satisfaction scores exceeded that reported in studies applying either communication models or clustering alone (41, 42). This may be attributed to the synergy between behavioral personalization and intelligent segmentation: DISC enables predictive, empathetic communication, while KANN-DBSCAN reveals complaint structures that inform targeted interventions and systematic quality improvement. By operationalizing psychological theory and advanced analytics into a practical framework, our study provides an evidence-based pathway toward more efficient, patient-centered, and trust-oriented hospital operations, addressing longstanding gaps in complaint management research.

Therefore, leveraging behavioral theory–based language models, particularly the DISC framework, in combination with advanced clustering methods enables not only descriptive but also prescriptive improvements in patient complaint management. By accurately categorizing unstructured complaint data and tailoring communication strategies to patients' behavioral styles, healthcare providers can anticipate conflict points, proactively address core concerns, and refine service workflows. This approach fosters trust-based doctor–patient relationships, reduces conflict escalation, and drives a transition toward more efficient, patient-centered, and intelligent hospital operations. Such personalization enhances communication efficiency, reduces escalation rates, and fosters a more empathetic patient experience. The result is not merely improved satisfaction scores, but a more resilient and trust-based doctor-patient relationship in high-pressure environments.

Furthermore, integrating language model theory into complaint management serves as a key innovation for modern healthcare systems. It facilitates fine-grained behavioral segmentation, allowing institutions to go beyond one-size-fits-all approaches. For example, identifying high-risk emotional cues early in the complaint process can trigger targeted interventions, thereby preventing conflict escalation or legal disputes.

### 5.1 Generalizability and broader applicability

The DISC-based language analysis framework is inherently adaptable, as it leverages universal principles of behavioral psychology and interpersonal communication. Many of the challenges identified in this study, such as misaligned expectations, emotional escalation, and inefficient complaint triage, are common across healthcare systems globally. By integrating DISC or similar personality-oriented models, hospitals can tailor communication strategies to individual patients, regardless of geographic or institutional context. The approach is scalable across different levels of healthcare—ranging from primary care clinics to tertiary hospitals—as long as staff are adequately trained in behavioral communication principles and digital tools are integrated into complaint intake workflows. In resource-limited settings, simplified DISC-mapping tools or rule-based sentiment classifiers may be sufficient to replicate some of the observed benefits.

### 5.2 Limitations and future improvement

However, several challenges remain. First, language models may struggle with nuanced, context-dependent emotions or culturally specific expressions. Second, while models can recommend communication strategies, frontline staff must still be trained to interpret and implement them effectively under stress.

Future research should focus on building closed-loop systems that not only analyze language and behavior in real time but also dynamically adjust response protocols. Longitudinal validation across different clinical departments and patient demographics will also be necessary to ensure generalizability and ethical robustness.

## 6 Conclusion

This study demonstrates that integrating the DISC behavioral language model with advanced clustering methods (KANN-DBSCAN) can substantially improve patient complaint management by enabling tailored, empathetic communication and data-driven identification of underlying complaint categories. These combined approaches led to measurable gains in patient satisfaction and reduced conflict escalation, addressing gaps that traditional complaint management methods often overlook. Our findings highlight the value of operationalizing behavioral theory alongside intelligent analytics to foster more patient-centered, efficient, and trust-oriented hospital operations. Future research should explore the scalability of this model across diverse healthcare settings and its long-term impact on service quality improvement.

## Data Availability

The raw data supporting the conclusions of this article will be made available by the authors, without undue reservation.
